# Textbook outcome following pancreaticoduodenectomy in elderly patients: age-stratified analysis and predictive factors

**DOI:** 10.1007/s13304-025-02130-3

**Published:** 2025-02-19

**Authors:** F. Mocchegiani, A. Benedetti Cacciaguerra, T. Wakabayashi, F. Valeriani, P. Vincenzi, F. Gaudenzi, D. Nicolini, G. Wakabayashi, M. Vivarelli

**Affiliations:** 1 Hepato-Pancreato-Biliary and Transplant Surgery, Department of Experimental and Clinical Medicine, Polytechnic University of Marche, 60126 Ancona, Italy; 2 Center for Advanced Treatment of Hepatobiliary and Pancreatic Diseases, Ageo Central General Hospital, Saitama, 362-8588 Japan; 3 Division of HPB and Abdominal Transplant Surgery, Department of Gastroenterology and Transplants, Azienda Ospedaliero-Universitaria Delle Marche, 60126 Ancona, Italy

**Keywords:** Surgery, Pancreaticoduodenectomy, Aged, Octogenarians, Outcomes

## Abstract

Despite advancements in pancreatic surgery, managing elderly patients undergoing pancreaticoduodenectomy (PD) remains challenging. Textbook Outcome (TO) serves as a benchmark for surgical success, but its relevance in elderly patients has not been well explored. This study aims to evaluate TO in elderly patients undergoing PD and identify predictors of TO failure. A retrospective analysis was conducted on elderly patients (≥ 70 years) who underwent PD between January 1, 2017, and December 31, 2023 in two international HPB centers. TO achievement rates were assessed and stratified by age groups (70–74, 75–79, ≥ 80). Uni- and multivariate logistic regression analyses were performed to identify risk factors for TO failure. Of 222 patients, 54.5% achieved TO after PD. TO rates decreased with age, with only 35.0% of octogenarians achieving TO, compared to 57.1% in those aged 70–74. Multivariate analysis revealed that age ≥ 80, an ASA score ≥ 2, and histopathologic types other than pancreatic ductal adenocarcinoma or distal cholangiocarcinoma were significant risk factors for failing to achieve TO. Nearly half of elderly patients achieved TO, with a lower likelihood in older age groups, particularly among octogenarians. Higher ASA scores were also associated with lower TO achievement. These findings underscore the importance of a comprehensive preoperative assessment, considering age, to optimize surgical outcomes in elderly patients undergoing PD.

## Introduction

Pancreaticoduodenectomy (PD) remains the cornerstone of treatment for various periampullary and pancreatic malignancies, despite being a complex surgical procedure associated with significant perioperative morbidity and mortality [1]. In high-volume centers, however, perioperative mortality is less than 4%, and studies have reported improvements in long-term oncological outcomes.

Despite advancements in surgical techniques, perioperative care, and patient selection, managing patients undergoing PD remains a significant challenge for pancreatic surgeons [1, 2].

With an aging population, there is increasing interest in understanding the outcomes of PD specifically in elderly patients. The true impact of age on perioperative outcomes in these patients is still not well defined. While some studies suggest that advanced age is a risk factor for higher postoperative complications and mortality [3, 4], others have failed to identify age as a significant risk factor in the postoperative course [5]. Nonetheless, the increased risk of major morbidity and mortality following PD appears to be more pronounced in elderly patients [6], likely due to reduced physiological reserve (homeostenosis), which diminishes their ability to tolerate major surgery and recover effectively, thereby increasing the risk of “failure to rescue” [7]. Beyond age, frailty may serve as a more accurate predictor of morbidity and mortality [8].

Textbook Outcome (TO) is a straightforward metric that encapsulates the best possible surgical outcome. For PD, van Roessel et al. [9] defined TO as the absence of postoperative pancreatic fistula (POPF), post-pancreatectomy hemorrhage (PPH), bile leakage (all International Study Group of Pancreas (ISGP) or International Study Group of Liver (ISGLS) grades B/C), any other complication classified as “major” (Dindo-Clavien ≥ III) [10]), in-hospital or 30-day mortality, and readmission within 30 days post-discharge. TO is considered achieved when all six parameters are fulfilled.

Understanding the nuances of achieving textbook outcome (TO) in elderly patients undergoing PD could inform clinical decision-making and optimize preoperative assessment. TO represents a composite measure, reflecting the most desirable surgical outcomes, and has been utilized in various surgical fields, including hepatopancreatobiliary (HPB) surgery. Achieving TO following PD is considered a benchmark for assessing the quality of surgical care and patient recovery. However, the applicability of TO in elderly patients undergoing PD remains understudied and warrants further investigation.

In this study, we aim to evaluate the rate of TO achievement following PD in elderly patients, stratified by age groups: 70–74 years, 75–79 years, and 80 years or older. Additionally, we will analyze the factors associated with the attainment of TO in this population.

## Methods

A retrospective analysis was conducted using a prospectively maintained database of all consecutive patients who underwent pancreaticoduodenectomy (PD) at in two international HPB centers […]. The study included patients aged 70 years or older who underwent PD between January 1, 2017, and December 31, 2023. The study was approved by the ethical committee of the Regione Marche (approval number 2024–4014).

### Study design

All consecutive elderly patients (age ≥ 70 years old) who underwent PD for all periampullary diseases were included in the analysis and divided in two groups, those who achieved Textbook Outcome (TO group) and those who did not achieved it (not-TO group).

The two groups were compared by analyzing baseline characteristics and intra- and postoperative outcomes. Uni- and multivariate logistic regression analyses were performed to assess the risk factors for not achieving TO.

Textbook Outcome (TO) is a composite measure that aims to encapsulate the entire surgical process into a single indicator. It reflects the ideal and most desirable surgical result, achieved when all prespecified TO criteria are met according to an all-or-nothing principle. In the context of pancreatic surgery, van Roessel et al. defined TO as the absence of postoperative pancreatic fistula (POPF), post-pancreatectomy hemorrhage (PPH), bile leakage (as categorized by the International Study Group of Pancreas (ISGP) or International Study Group of Liver (ISGLS) grades B/C), the occurrence of any major complication (Dindo-Clavien grade III or higher), in-hospital or 30-day mortality, and readmission within 30 days post-discharge. TO is deemed achieved only when all six specified parameters are fulfilled.

Baseline patient characteristics included age and American Society of Anesthesiologists (ASA) score. For the purpose of age-stratified analysis, age was treated both as a continuous variable and categorized into three groups: 70–74 years, 75–79 years, and ≥ 80 years.

Frailty is a prevalent condition among elderly patients. To assess frailty in our study population, we calculated the Modified Frailty Index (mFI) score [11] using information from patients’ medical histories and records. While the mFI score is not intended to be a dichotomous variable, a cutoff of 0.27 was used to classify patients as “frail” or “pre-frail,” based on a comprehensive literature review.

PD procedures were performed using either a pylorus-preserving technique or a subtotal stomach-preserving approach and standard lymph-node dissection according to ISGPS definition. Both open and minimally invasive approaches (MIPS) followed the same steps for the demolitive and reconstructive phases. The preferred technique for pancreatic anastomosis was Blumgart-style duct-to-mucosa pancreatico-jejunostomy. Hepaticojejunostomy was used for biliary reconstruction, performed in an end-to-side fashion. A pylorus-jejunostomy was placed 50–60 cm distal to the biliary anastomosis on the same loop.

Details about the procedure, including type of access, operative time, and intraoperative estimated blood loss, were recorded. Information regarding pancreatic gland characteristics, such as texture (soft or hard) and pancreatic duct diameter, was also documented. Both early and late postoperative courses were followed. Specific complications related to pancreatic surgery were of particular interest in this study: postoperative pancreatic fistula (POPF), postoperative biliary fistula (POBF), and post-pancreatectomy hemorrhage (PPH) were defined according to the International Study Group on Pancreatic Surgery (ISGPS) and International Study Group of Liver Surgery (ISGLS) criteria [12–14].

### Preoperative assessment

An in-depth review of patient histories was conducted to identify any clinical manifestations at disease onset. Additionally, patients presenting with jaundice or elevated serum bilirubin levels were assessed to determine the need for biliary drainage as “bridge therapy” prior to attempting radical surgical resection. In our study, more than half of the patients underwent treatment after being referred from low-volume centers or rural hospital. Although there were no standardized protocols for the palliation of jaundice, when patients were initially evaluated at the two participating centers and exhibited jaundice (defined as a pre-operative serum bilirubin level greater than 7.5 mg/dl), they were deemed eligible for the PBD procedure. PBD was typically performed through endoscopic retrograde cholangiopancreatography (ERCP) with the placement of a plastic or metallic-covered stent. When this approach was not feasible, percutaneous trans-hepatic biliary drainage (PTBD) was employed. Resectability status was determined using the 2024 National Comprehensive Cancer Network (NCCN) guidelines and was classified as resectable (R), borderline resectable (BR), or locally advanced (LA) [15]. Preoperative diagnostic imaging (CT scan and/or MRI) was used to assess this status. The majority of resectable tumors underwent up-front surgery, with neoadjuvant therapy (NAT) prioritized for patients presenting a combination of the standard radiologic criteria for resectability and high-risk factors (elevated carbohydrate antigen 19–9 levels, large tumor size, suspected metastases in regional lymph nodes, significant weight loss, and pain). All borderline resectable/locally advanced (BR/LA) tumors received NAT followed by surgical resection. At our institutions, the NAT regimen and cycle duration were determined by the multidisciplinary team (MDT) based on the patients’ performance status, symptoms, tumor markers, and radiographic images after each treatment course. Gemcitabine with tegafur/gimeracil/oteracil (GS), albumin-bound paclitaxel combined with tegafur/gimeracil/oteracil (AS), and modified 5-fluorouracil, leucovorin, irinotecan, and oxaliplatin (mFOLFIRINOX) has been recommended as the standard first-line neoadjuvant treatment. Other regimens were gemcitabine combined with albumin-bound paclitaxel or gemgitabine alone. Surgical resection is typically performed within 4 weeks following the last cycle of neoadjuvant chemotherapy.

Data regarding the pancreaticoduodenectomy specimen were collected from the pathology report.

### Statistical analysis

Continuous data were reported as the mean with standard deviation and were compared using a two-sided Student t test for normally distributed parameters. Tests for normality were performed using Kolmogorov–Smirnov and Shapiro–Wilk tests. Continuous, not normally distributed variables were described as median (IQR) and compared using the Wilcoxon–Mann–Whitney test. Comparisons between groups for categorical variables were performed using the Chi-square test with Yates’ correction or Fisher exact test, when appropriate. To identify variables that were independent predictors for not achieving TO univariate logistic regression analysis was performed across the whole cohort. Subsequently, multivariate logistic regression was conducted for factors with statistical relevance (variables with a P-value < 0.05 on univariate analysis). Results of logistic regression were reported as odds ratios (OR) and 95% CI. Statistical analysis was performed using the Statistical Package for Social Sciences (Version 27.0; SPSS Inc, Chicago, IL, USA).

## Results

Among a total of 669 patients who underwent pancreatic resections at 2 centers (Ancona, Italy and Ageo, Japan), 222 patients met the inclusion criteria. These patients were divided into 2 groups: 121 who achieved Textbook Outcome (TO group) and 101 who did not meet the criteria for TO (non-TO group).

### Preoperative and intraoperative characteristics

The baseline characteristics of patients are summarized in Table 1. Age was reported as a continuous variable and categorized into age groups. The median age was similar between the two groups; however, when stratifying by age groups, younger patients were more prevalent in the TO group, while octogenarians were more represented in the non-TO group (*p* = 0.023).

**Table 1:** baseline characteristics for overall patient population and for Non-TO and TO groups

Baseline characteristics	Overall (*n* = 222)	Not–TO group (*n* = 101)	TO group (*n* = 121)	*p* value
Sex, Male (%)	137 (61.7)	65 (64.4)	72 (71.3)	0.459
Age, years (IQR)	76.0 (73.0–79.0)	76.0 (73.0–80.0)	76.0 (73.5–80.0)	0.914
Age (%)				**0.023**
70–74	77 (34.7)	31 (30.7)	46 (38.0)	
75–79	105 (47.3)	44 (43.6)	61 (50.4)	
≥ 80	40 (18.0)	26 (25.7)	14 (11.6)	
BMI (IQR)	22.9 (21.0–24.7)	23.1 (21.1–25.0)	22.8 (20.8–24.7)	0.329
ASA (%)				**0.003**
I	19 (8.6)	5 (5.0)	14 (11.6)	
II	154 (69.4)	64 (63.3)	90 (74.4)	
III	49 (22.1)	32 (31.7)	17 (14.0)	
mFI (IQR)	0.09 (0.00–0.18)	0.09 (0.00–0.18)	0.09 (0.09–0.18)	**0.010**
mFI (%)				0.101
Pre-frail	141 (63.5)	70 (69.3)	71 (58.7)	
Frail	81 (36.5)	31 (30.7)	50 (41.3)	
Symptoms (%)	98 (44.1)	46 (45.5)	52 (43.0)	0.701
Jaundice	54 (24.3)	25 (20.7)	29 (24.0)	0.892
Pain	18 (8.1)	11 (10.9)	7 (5.8)	0.165
Pancreatitis	2 (2.7%)	2 (2.0)	0 (0.0)	0.157
Weight loss	11 (5.0)	8 (7.9)	3 (2.5)	0.063
Diabetes	79 (35.6)	26 (25.7)	53 (43.8)	**0.005**
Biliary drainage (%)	116 (52.3)	55 (54.5)	61 (50.4)	0.317
Endoscopic	114 (51.4)	55 (54.5)	59 (48.8)	0.398
Percutaneous	2 (0.9)	0 (0.0)	2 (1.7)	0.194
WBC, × 10^9^/L (IRQ)	6.0 (5.0–7.1)	6.0 (5.0–7.7)	6.0 (4.9–7.0)	0.532
Haemoglobin, g/dL (IQR)	12.0 (10.6–13.2)	12.2 (10.9–13.4)	12.0 (10.5–13.0)	0.246
Platelet, × 10^9^/L (IQR)	214.5 (180.0–275.3)	225.0 (180.5–283.5)	212.5 (178.5–257.0)	0.176
Total bilirubin, mg/dL (IQR)	0.8 (0.5–1.5)	0.9 (0.5–1.9)	0.8 (0.6–1.5)	0.497
Albumin [g/L], (IQR)	3.0 (3.0–4.0)	3.0 (3.0–4.0)	3.1 (3.0–4.0)	0.863
AST, UI/L (IQR)	25.0 (18.8–47.5)	25.0 (17.5–55.0)	25.0 (19.5–42.5)	0.758
ALT UI/L (IQR)	27.5 (17.0–70.0)	26.0 (16.5–67.0)	28.0 (17.0–73.0)	0.771
Creatinine, mg/dL (IQR)	0.87 (0.69–1.00)	0.86 (0.69–1.00)	0.90 (0.68–1.00)	0.974
CA 19.9, UI/L (IQR)	32.5 (7.0–171.5)	32.5 (9.3–139.3)	32.5 (6.0–249.3)	0.906
Resectability status (%)				0.694
R	201 (90.5)	90 (89.1)	111 (91.7)	
BR	18 (8.1)	9 (8.9)	9 (7.4)	
LA	3 (1.4)	2 (2.0)	1 (1.0)	
Neoadjuvant chemotherapy	32 (14.4)	13 (12.9)	19 (15.7)	0.550

*ALT* alanine aminotransferase, *ASA* American Society of Anesthesiologists, *AST* aspartate aminotransferase, *BMI* body mass index, *BR* borderline resectable, *IQR* interquartile range (25°–75°), *LA* locally advanced, *mFI* modified Frailty index, *R* resectable, *TO* textbook outcome, *WBC* white blood cells

American Society of Anesthesiologists (ASA) grade III was more common in the non-TO group (31.7% vs 14.0%, *p* = 0.003), while most ASA I patients were in the TO group (11.6% vs 5.0%). The percentage of frail patients was higher in the TO group compared to the non-TO group, but this difference did not reach statistical significance (41.3% vs 30.7%, *p* = 0.101). Regarding diabetes history, more patients with diabetes were in the TO group compared to the non-TO group (43.8% vs 25.7%; *p* = 0.005).

Perioperative characteristics are detailed in Table 2. Regarding surgical approach, 149 were performed using an open technique, and 73 were minimally invasive (18 laparoscopic resections, 8.1%, and 55 robotic interventions, 24.8%). Five cases of laparoscopic procedures required an unplanned conversion to open surgery due to technical difficulty mainly during reconstructive phase. 2 conversions were associated with TO achievement, while in the other 3 cases, due to postoperative complications, patients failed to reach TO. Looking more in details, while open PD was performed more frequently (67.1%), Robotic PD allowed to achieve a higher rate of TO (28.1%) in comparison to the open and laparoscopic approach (66.1% and 5.8%, respectively), but this difference fail to reach statistical significance.

**Table 2:** overview of perioperative characteristics in overall patient population, in Not-TO and TO group

Perioperative characteristics	Overall (*n* = 222)	Not–TO group (*n* = 101)	TO group (*n* = 121)	*p* value
Access (%)				0.224
Open	149 (67.1)	69 (68.3)	80 (66.1)	
Laparoscopic	18 (8.1)	11 (10.9)	7 (5.8)	
Robotic	55 (24.8)	21 (20.8)	34 (28.1)	
Operating time, min (IQR)	450 (380–565)	457 (389–565)	445 (375–565)	0.689
Intraoperative blood loss, ml (IQR)	334.0 (194.0–503.0)	334.0 (200.0–600.0)	334.0 (154.0–500.0)	0.260
Pancreatic duct diameter, mm (IQR)	3.0 (2.0–5.0)	3.0 (2.0–3.8)	4.0 (2.5–6.3)	**0.002**
Pancreatic duct diameter (%)				**0.012**
< 3 mm	85 (38.5)	51 (50.5)	34 (28.1)	
≥ 3 mm	137 (61.5)	50 (49.5)	87 (71.9)	
Pancreatic texture (%)				0.054
Soft	124 (56.0)	69 (68.3)	55 (45.5)	
Hard	98 (44.0)	40 (39.6)	58 (47.9)	
Postoperative hospital stays, days (IQR)	17 (12–27)	25 (15–38)	14 (10–19)	** < 0.001**
Adjuvant chemotherapy (%)	77 (34.7)	28 (27.7)	49 (40.5)	0.104
Overall survival (IQR)	18.3 (7.9–38.4)	17.8 (7.6–42.5)	18.4 (8.7–33.5)	0.921

*IQR* interquartile range (25°–75°), *TO* Textbook Outcome

Pancreatic duct diameter was measured intraoperatively, and its median value was higher for TO group (*p* = 0.002). This difference was also confirmed when pancreatic duct diameter was categorized in two groups (*p* = 0.012): < 3 mm vs ≥ 3 mm. Looking at the pancreatic texture: 124 patients (56.0%) had soft pancreas [69 (68.3%) in not-TO group vs 55 (45.5%) in TO group] while 98 patients (44.0%) had hard pancreas [40 (39.6%) in not-TO group vs 58 (47.9%) in TO group] but this value nearly fail to reach statistical significance (*p* = 0.054).

### Histopathological characteristics

Among the histopathological data collected (Table 3), the pT stage was significantly different between the two groups (*p* = 0.035). Specifically, pT4 tumors were diagnosed exclusively in the non-TO group (three patients, 100% vs 0 in the TO group). Ductal adenocarcinoma was the most frequent diagnosis, occurring in 130 patients (58.5%), followed by cholangiocarcinoma in 34 patients (15.2%) and other histotypes in 58 patients (26.2%). The latter group included cases of mucinous intraductal papillary adenoma, mucinous intraductal tubular adenoma, mucinous carcinoma, and neuroendocrine tumors (NET). While no significant difference emerged from the analysis, ductal adenocarcinoma was more common in the TO group (65.3% vs 50.5%), whereas other histopathologic subtypes were more frequent in the non-TO group (33.7% vs 19.8%). The incidence of cholangiocarcinoma was similar in both groups.

**Table 3:** Histopathological features in overall patient population, in Not-TO and TO group

Hystological features	Overall (*n* = 222)	Not-TO group (*n* = 101)	TO group (*n* = 121)	*p* value
Histopathological type (%)				0.120
Adenocarcinoma	130 (58.5)	51 (50.5)	79 (65.3)	
Cholangiocarcinoma	34 (15.2)	16 (15.8)	18 (14.9)	
Other	58 (26.2)	34 (33.7)	24 (19.8)	
Lesion diameter, mm (IQR)	26.0 (20.0–38.0)	20.0 (20.0–32.5)	30.0 (20.0–40.0)	0.548
T (%)				**0.035**
is	24 (11.0)	7 (6.9)	17 (14.0)	
1	40 (17.8)	26 (25.7)	14 (11.6)	
2	93 (41.8)	37 (36.6)	56 (46.3)	
3	62 (28.1)	28 (27.8)	34 (28.1)	
4	3 (1.4)	3 (3.0)	0 (0.0)	
N (%)				0.451
0	89 (40.0)	41 (40.6)	48 (39.6)	
1	84 (37.9)	40 (39.6)	44 (36.4)	
2	49 (22.1)	20 (19.8)	29 (24.0)	
Staging (%)				0.293
0	20 (9.0)	6 (5.9)	14 (11.6)	
IA	34 (14.6)	20 (19.8)	14 (11.6)	
IB	18 (8.3)	8 (7.9)	10 (8.3)	
IIA	15 (6.9)	6 (5.9)	9 (7.4)	
IIB	77 (34.7)	37 (36.6)	40 (33.1)	
III	49 (22.2)	15 (14.9)	34 (28.1)	
IV	9 (4.2)	8 (7.9)	1 (0.8)	
Grading (%)				0.929
1	74 (33.3)	32 (31.7)	42 (34.7)	
2	95 (42.9)	45 (44.6)	50 (41.3)	
3	53 (23.8)	24 (23.8)	29 (24.0)	
Histological margin (%)				0.129
R0	125 (56.2)	68 (67.3)	57 (47.1)	
R1	97 (43.8)	33 (32.7)	64 (52.9)	
Perineural invasion (%)	88 (39.6)	41 (40.6)	47 (38.8)	0.486
Lymphovascular invasion (%)	72 (32.4)	33 (32.7)	39 (32.2)	0.659

I*QR* interquartile range (25°–75°), *TO* Textbook Outcome

None of the patients had chronic pancreatitis as the primary indication for surgery; however, some individuals who underwent surgery for PDAC had experienced episodes of pancreatitis in their medical history.

### Postoperative outcomes

**Fig. 1:**
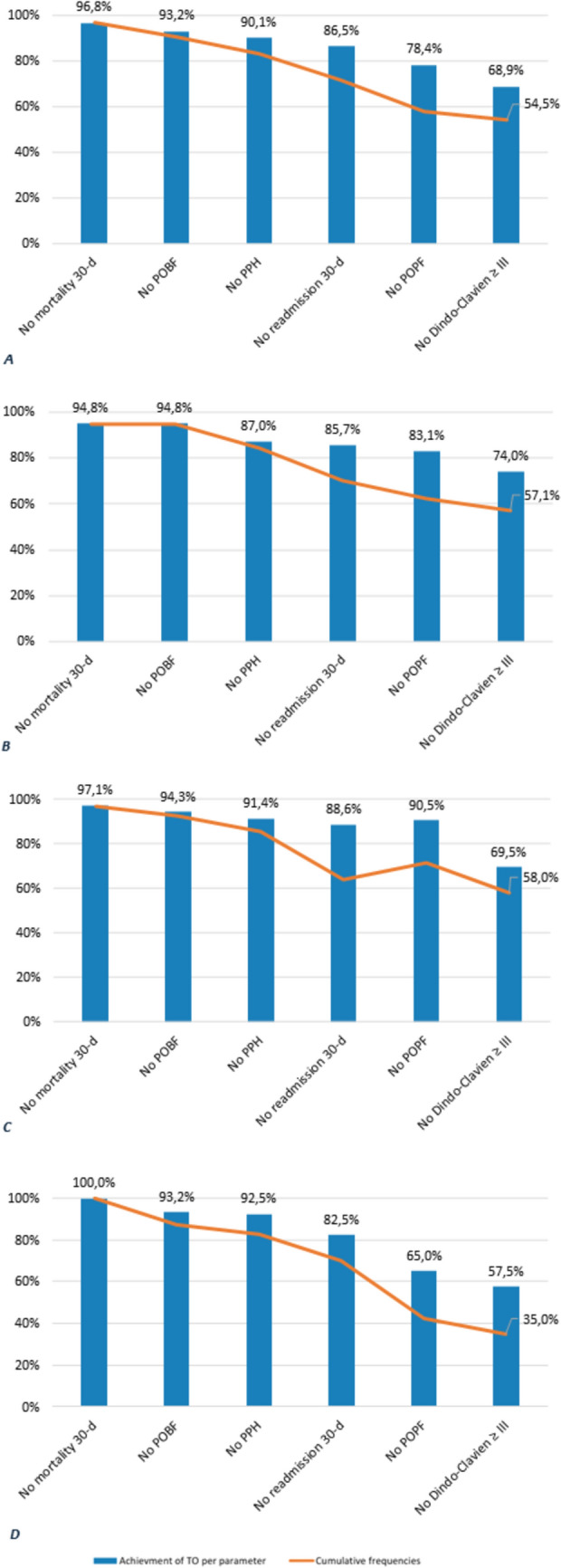
Frequencies for achievement of TO per parameter and cumulative frequencies in (**A**) overall patient population, (**B**) 70–74 years old patient population, (**C**) 75–79 years old patient population and (D) ≥ 80 years old patient population. The overall TO achievement rate was 54.5%, which decreased across age groups, with only 35.0% of octogenarians achieving TO. In each cohort, the most frequently met criterion was “No mortality within 30 days,” while the least frequently met was “No Dindo-Clavien ≥ III.” Notably, in the octogenarian cohort, nearly half of the patients (42.5%) experienced major complications

The prevalence of the six individual outcome metrics included in the definition of TO is displayed in Table 4. A total of 121 patients (54.5%) achieved all six criteria, thereby fulfilling the definition of Textbook Outcome. The absolute and cumulative frequencies of TO in the overall population and in each age group are shown in Fig. 1A, B, C, and D.

**Table 4:** rates of post-operative complications integrated in TO in overall patient population and stratified in each age groups

Post-operative complications	Overall (*n* = 222)	70–74 years old group (*n* = 77)	75–79 years old group (*n* = 105)	≥ 80 years old group (*n* = 40)
POPF (%)	48 (21.6)	13 (16.9)	21 (20.0)	14 (35)
POBF (%)	15 (6.8)	4 (5.2)	6 (5.7)	5 (6.8)
PPH (%)	22 (9.9)	10 (13.0)	9 (8.6)	3 (7.5)
Major complications (%)	69 (31.1)	20 (26.0)	32 (30.5)	17 (42.5)
30 day readmission (%)	30 (13.5)	11 (14.3)	12 (11.4)	7 (17.5)
30 days mortality (%)	7 (3.2)	4 (5.2)	3 (2.9)	0 (0.0)

*POBF* Post-operative biliary fistula, *POPF* Post-operative pancreatic fistula, *PPH* Post-pancreatectomy haemorrhage

### Risk factors for not achieving textbook outcome

Univariate analysis revealed that octogenarian patients were at a significantly higher risk for not achieving TO [OR 2.756 (95% CI 1.246–6.093); *p* = 0.012]. Other preoperative factors associated with a lower rate of TO included ASA II grade [OR 1.991 (95% CI 0.683–5.806); *p* = 0.207] and ASA III grade [OR 5.271 (95% CI 1.622–17.126); *p* = 0.006]. Additionally, a histopathological diagnosis other than pancreatic adenocarcinoma or cholangiocarcinoma was a predictor for not achieving TO [OR 2.105 (95% CI 1.014–4.369); *p* = 0.046]. A smaller pancreatic duct diameter was also predictive of not reaching TO [OR 2.500 (95% CI 1.211–5.160); *p* = 0.013]. Conversely, diabetes had a protective effect on achieving TO [OR 0.445 (95% CI 0.251–0.789); *p* = 0.006].

Multivariate analysis identified the following independent risk factors for not achieving TO: age ≥ 80 [OR 15.949 (95% CI 1.641–154.956); *p* = 0.017], ASA II [OR 4.332 (95% CI 1.148–16.343); *p* = 0.030] and ASA III [OR 4.992 (95% CI 1.094–22.777); *p* = 0.038), and histopathological diagnosis other than pancreatic adenocarcinoma or cholangiocarcinoma [OR 3.167 (95% CI 1.084–9.256); *p* = 0.035] (Table 5).

**Table 5:** Univariate and Multivariate analysis for risk factors for not achieving TO

	Univariate analysis		Multivariate analysis	
Factor	OR (95% CI)	*P*	OR (95% CI)	*P*
Sex, male	1.229 (0.712–2.120)	0.459		
Age	1.004 (0.942–1.069)	0.910		
Age				
70–74	Ref		Ref	
75–79	1.070 (0.589–1.947)	0.824	1.407 (0.552–3.589)	0.474
≥ 80	2.756 (1.246–6.093)	**0.012**	15.949 (1.641–154.956)	**0.017**
BMI	1.041 (0.950–1.141)	0.387		
mFI				
Pre-Frail	Ref			
Frail	1.590 (0.911–2.774)	0.102		
Biliary drainage	1.274 (0.749–2.166)	0.371		
ASA				
I	Ref		Ref	
II	1.991 (0.683–5.806)	0.207	4.332(1.148–16.343)	**0.030**
III	5.271 (1.622–17.126)	**0.006**	4.992 (1.094–22.777)	**0.038**
Symptoms	1.110 (0.652–1.889)	0.701		
Diabetes	0.445 (0.251–0.789)	**0.006**	0.400 (0.134–1.188)	0.099
Resectability status R BR LA				
	Ref			
	1.233 (0.470–3.237)	0.670		
	2.467 (0.220–27.643)	0.464		
Neoadjuvant chemotherapy	0.793 (0.371–1.697)	0.550		
Access				
Open	Ref			
Laparoscopic	1.822 (0.670–4.9557)	0.240		
Robotic	0.716 (0.381–1.347)	0.301		
Histopathological type				
PDAC	Ref		Ref	
CCA	1.583 (0.634–3.734)	0.341	3.844 (0.845–17.492)	0.082
Other	2.105 (1.014–4.369)	**0.046**	3.167 (1.084–9.256)	**0.035**
Lesion diameter	0.990 (0.972–1.008)	0.254		
Operative time	1.001 (0.999–1.002)	0.525		
Intraoperative blood loss	1.000 (1.000–1.000)	0.908		
Pancreatic duct diameter				
≥ 3 mm	Ref		Ref	
< 3 mm	2.500 (1.211–5.160)	**0.013**	1.545 (0.576–4.143)	0.388
Pancreatic Texture				
Hard	Ref			
Soft	1.835 (0.955–3.525)	0.068		

*ASA* American Society of Anesthesiologists, *BMI* body mass index, *BR* borderline resectable, *CCA* cholangiocarcinoma, *CI* confidence interval, *IQR* interquartile range (25°–75°), *LA* locally advanced, *mFI* modified Frailty index, *OR* odds ratio, *PDAC* pancreatic ductal adenocarcinoma, *R* resectable, *TO* textbook outcome

## Discussion

To our knowledge, this is the first study to evaluate the rates of Textbook Outcome (TO) achievement following pancreaticoduodenectomy (PD) in elderly patients. More specifically, this study underscores the importance of preoperative patient selection and its impact on surgical outcomes by identifying risk factors associated with not achieving TO. The insights from this study may aid in risk stratification, patient counseling, and the development of tailored interventions aimed at improving outcomes in this vulnerable patient population.

Modern medicine is shifting toward a personalized approach, considering each patient as an individual and providing the best treatment option with minimal “human expense.” This philosophy aligns perfectly with the need to improve the quality of procedures by reducing complications and ensuring favorable oncological outcomes. In this context, TO has emerged as a breakthrough. TO is a straightforward metric that encapsulates the ideal surgical outcome for a range of procedures, from colorectal to gastrointestinal surgeries [16, 17], and more recently, HPB surgeries [9, 18]. Moreover, TO has been adopted as a benchmark for clinical practice in pancreatic surgery. By comparing local outcomes with national databases, TO provides a continuous measure of performance and valuable feedback for quality improvement [19]. Pancreaticoduodenectomy (PD) is the gold standard treatment for resectable periampullary diseases and remains the only curative option for pancreatic malignancies. However, it is still a high-risk surgical procedure [1]. Since 2020, when van Roessel et al. first introduced the TO definition for PD, several studies have focused on measuring TO achievement, with success rates ranging from 56 to 69%. However, the potential impact of advanced age on TO achievement has not been specifically assessed [20, 21]. PD is feasible in elderly patients but is associated with higher complication rates, even when performed by experienced surgeons in high-volume centers [1, 3]. In this study, we observed a stepwise decrease in TO achievement across different age groups, with octogenarians achieving the lowest rate (35.0%). This finding was confirmed in our multivariate analysis, where age ≥ 80 years was an independent risk factor for not achieving TO [OR 2.756 (95% CI 1.246–6.093); *p* = 0.012]. Of interest, in octogenarians experienced 30-day mortality rate of 0%, which is somehow surprising in this population. However, these data may underscore the challenges faced by octogenarian patients in achieving desired clinical outcomes compared to TO.

Beyond chronological age, biological age—reflected by frailty—may be a better predictor of adverse outcomes. Recent evidence highlights a progressive increase in major postoperative complications and mortality in frail patients [8]. We hypothesize that frailty might also be associated with a lower likelihood of achieving TO. Surprisingly, our results revealed that frail patients were more likely to be in the TO group. It is important to note that our study population was highly selected, with characteristics differing from those in studies evaluating the predictive value of the Modified Frailty Index (mFI). The mFI has been validated as a predictor of outcomes in younger patient populations, but only a few studies have explored its predictive value in pancreatic surgery [8]. Moreover, mFI score frames frailty as the accumulation of multiple deficits [22]. Another notable finding in this study was the correlation between ASA grade and TO achievement. Indeed, many comorbidities included in the ASA score are highly prevalent in elderly patients and have a detrimental impact on postoperative outcomes, as highlighted by a recent meta-analysis by Tan et al. This analysis showed a sharp increase in postoperative complications in patients with an ASA score ≥ III [23]. In our study, an ASA score ≥ II was a predictor of not achieving TO, which aligns with previous research indicating that ASA score is a key factor in predicting lower TO rates [9]. While the ASA score and mFI score both predict postoperative risk based on preoperative comorbidities, their correlation has been questioned. For instance, in urologic surgery, Serretta et al. showed that the ASA score and mFI score were linked only for low-risk procedures, while this correlation was absent for high-risk surgeries. Specifically, in patients with an ASA score ≥ III, only 22% were classified as frail [24]. As PD is a high-risk procedure, it is not surprising that the ASA and mFI scores may not be as closely correlated in this context.

In terms of comorbidities, diabetes deserves special mention. This condition is highly prevalent in the general population, especially in elderly individuals, and it can increase perioperative risk, as it is part of the ASA score. The impact of diabetes on postoperative outcomes after pancreatic resection remains debated in the literature. While some studies found no correlation between diabetes and postoperative complications [25], others, such as the study by Chong et al., highlighted that diabetes is associated with a tougher pancreatic texture [26]. This might explain the lower incidence of pancreatic fistula (POPF) in diabetic patients. In our study, diabetes was linked to a higher chance of achieving TO in univariate analysis (43.8% vs 25.7%; OR 0.445 [95% CI 0.251–0.789]; *p* = 0.006). Unfortunately, this association was not confirmed in the multivariate analysis.

The pancreatic stump is often considered the “Achilles’ heel” of pancreatic surgery, as numerous studies have shown a correlation between soft pancreatic tissue and narrow pancreatic ducts with an increased risk of POPF [9, 27]. Our study mirrors these findings, but interestingly, pancreatic stump characteristics were not confirmed as significant risk factors for not achieving TO in multivariate analysis. The features of the pancreatic stump interact with other factors, such as age ≥ 80, ASA score ≥ II, and non-pancreatic adenocarcinoma histology, all of which contribute to a lower likelihood of achieving TO. Although each of these variables independently affects postoperative outcomes, advanced age and poor performance status have a more pronounced impact. PD is one of the most challenging general surgeries, and its complexity often leads to a cascade of complications, especially in elderly patients with limited functional reserve. Indeed, in our cohort, most of the adverse outcomes preventing TO achievement were non-surgical complications classified as Clavien–Dindo ≥ III.

Finally, patients with periampullary neoplasms, including those with papillary, duodenal, cystic, or islet cell pathologies, are at a higher risk of developing POPF. This study confirms that histologies other than pancreatic adenocarcinoma (PDAC) or cholangiocarcinoma are associated with a lower rate of TO achievement [9, 27]. When periampullary masses are diagnosed as benign neoplasms or non-cancerous diseases, they often have an excellent prognosis and long-term survival after surgery. However, these histotypes are linked to a significantly higher risk of POPF, worsening the postoperative course and increasing mortality [27, 28]. In contrast, PDAC has a poor prognosis, but it may serve as a “protective” factor against POPF, as its growth often leads to obstruction and dilation of the pancreatic duct, which reduces the risk of fistula formation and enhances the likelihood of achieving TO.

Our findings highlight key risk factors for not achieving TO following PD in elderly patients, which may be valuable for multidisciplinary tumor boards in clinical decision-making. Major surgeries, like pancreatic resections, require a delicate balance between a patient’s frailty and the potential benefits of surgery, especially when the risks involved are high. As the saying goes in HPB surgery, “The liver forgives you, but the pancreas does not.” The liver has remarkable regenerative abilities, while the pancreas is less forgiving and prone to complications when damaged. In this context, careful preoperative selection of elderly patients for PD is crucial. Age alone should not be a reason to exclude patients from surgery, but PD may not be the best choice for octogenarians with periampullary diseases. Similarly, special attention should be given to patients with an ASA score ≥ II or a histopathological diagnosis other than PDAC or cholangiocarcinoma.

There are several limitations to this study. The retrospective design and relatively small sample size must be acknowledged. First, not all patients diagnosed with periampullary neoplasia have resectable disease, and this study did not employ an intention-to-treat design. Only patients who underwent PD were included, excluding those who were not candidates for surgery. Second, while PD is feasible in elderly patients, it is associated with serious complications, so careful selection is essential. Another limitation is the difference in disease epidemiology between Western and Eastern countries, such as the Italian and Japanese populations. However, both Italy and Japan are known for having some of the highest life expectancies in the world, and this study represents one of the largest experiences with elderly patients undergoing PD.

## Conclusion

In conclusion, our study identified several key factors associated with the achievement of Textbook Outcome (TO) following pancreatic resections, including age, ASA grade, and tumor histopathology. These findings emphasize the critical importance of a thorough preoperative assessment and individualized surgical planning in optimizing outcomes for patients undergoing pancreatic surgery, particularly elderly individuals. Future research should focus on the prospective validation of these predictors and the development of tailored interventions aimed at enhancing surgical success and minimizing complications. By refining patient selection and surgical strategies, we can improve the overall effectiveness and safety of pancreatic resections, even in high-risk populations.

## References

[1] Cameron JL, Riall TS, Coleman J, Belcher KA (2006) One thousand consecutive pancreaticoduodenectomies. Ann Surg 244(1):10–15 16794383 10.1097/01.sla.0000217673.04165.ea PMC1570590

[2] Fong Y, Gonen M, Rubin D, Radzyner M, Brennan MF (2005) Long-term survival is superior after resection for cancer in high-volume centers. Ann Surg 242(4):540–547 16192814 10.1097/01.sla.0000184190.20289.4b PMC1402350

[3] Kim SY, Weinberg L, Christophi C, Nikfarjam M (2017) The outcomes of pancreaticoduodenectomy in patients aged 80 or older: a systematic review and meta-analysis. HPB 19(6):475–482 28292633 10.1016/j.hpb.2017.01.018

[4] Prashant S, Jonathan T, Mauricio S, James S, Peter D (2012) Advanced age is a risk factor for post-operative complications and mortality after a pancreaticoduodenectomy: a meta-analysis and systematic review. HPB 14(10):649–657 22954000 10.1111/j.1477-2574.2012.00506.x PMC3461370

[5] Liang DH, Shirkey BA, Rosenberg WR, Martinez S (2016) Clinical outcomes of pancreaticoduodenectomy in octogenarians: a surgeon’s experience from 2007 to 2015. J Gastrointest Oncol 7(4):540–546 27563443 10.21037/jgo.2016.03.04 PMC4963361

[6] Kane RL, Shamliyan T, Talley K, Pacala J (2012) The association between geriatric syndromes and survival. J Am Geriatr Soc 60(5):896–904 22568483 10.1111/j.1532-5415.2012.03942.x

[7] Tamirisa NP, Parmar AD, Vargas GM, Mehta HB, Molly Kilbane E, Hall BL et al (2016) Relative contributions of complications and failure to rescue on mortality in older patients undergoing pancreatectomy. Ann Surg 263(2):385–391 25563871 10.1097/SLA.0000000000001093 PMC5404345

[8] Mogal H, Vermilion SA, Dodson R, Hsu FC, Howerton R, Shen P et al (2017) Modified frailty index predicts morbidity and mortality after pancreaticoduodenectomy. Ann Surg Oncol 24(6):1714–1721 28058551 10.1245/s10434-016-5715-0 PMC7064816

[9] van Roessel S, Mackay TM, van Dieren S, van der Schelling GP, Nieuwenhuijs VB, Bosscha K et al (2020) Textbook outcome: nationwide analysis of a novel quality measure in pancreatic surgery. Ann Surg 271(1):155–162 31274651 10.1097/SLA.0000000000003451

[10] Dindo D, Demartines N, Clavien PA (2004) Classification of surgical complications: a new proposal with evaluation in a cohort of 6336 patients and results of a survey. Ann Surg 240(2):205–213 15273542 10.1097/01.sla.0000133083.54934.ae PMC1360123

[11] Velanovich V, Antoine H, Swartz A, Peters D, Rubinfeld I (2013) Accumulating deficits model of frailty and postoperative mortality and morbidity: Its application to a national database. J Surg Res 183(1):104–110 23415494 10.1016/j.jss.2013.01.021

[12] C. Bassi, G. Marchegiani, C. Dervenis, M. Sarr, M. Abu Hilal, M. Adham, et al., ‘The 2016 update of the International Study Group (ISGPS) definition and grading of postoperative pancreatic fistula: 11 Years After’, Mar. 01, 2017, Mosby Inc. 10.1016/j.surg.2016.11.014 28040257

[13] Koch M, Garden OJ, Padbury R, Rahbari NN, Adam R, Capussotti L et al (2011) Bile leakage after hepatobiliary and pancreatic surgery: a definition and grading of severity by the international study group of liver surgery. Surgery 149(5):680–688 21316725 10.1016/j.surg.2010.12.002

[14] Wente MN, Veit JA, Bassi C, Dervenis C, Fingerhut A, Gouma DJ et al (2007) Postpancreatectomy hemorrhage (PPH)-an international study group of pancreatic surgery (ISGPS) definition. Surgery 142(1):20–25 17629996 10.1016/j.surg.2007.02.001

[15] National Comprehensive Cancer Network, ‘NCCN clinical practice guidelines in oncology - Pancreatic Adenocarcinoma. V.2.2024’.

[16] Kolfschoten NE, Kievit J, Gooiker GA, van Leersum NJ, Snijders HS, Eddes EH et al (2013) Focusing on desired outcomes of care after colon cancer resections; hospital variations in “textbook outcome.” Euro J Surg Oncol (EJSO) 39(2):156–163 10.1016/j.ejso.2012.10.007 23102705

[17] van der Kaaij RT, de Rooij MV, van Coevorden F, Voncken FEM, Snaebjornsson P, Boot H et al (2018) Using textbook outcome as a measure of quality of care in oesophagogastric cancer surgery. Br J Surg 105(5):561–569 29465746 10.1002/bjs.10729

[18] Görgec B, Benedetti Cacciaguerra A, Lanari J, Russolillo N, Cipriani F, Aghayan D et al (2021) Assessment of textbook outcome in laparoscopic and open liver surgery. JAMA Surg 156(8):e212064 34076671 10.1001/jamasurg.2021.2064 PMC8173471

[19] Nicholas E, van Roessel S, de Burlet K, Hore T, Besselink MG, Connor S (2021) Using textbook outcomes to benchmark practice in pancreatic surgery. ANZ J Surg 91(3):361–366 33475226 10.1111/ans.16555

[20] Suurmeijer JA, Henry AC, Bonsing BA, Bosscha K, van Dam RM, van Eijck CH et al (2023) Outcome of pancreatic surgery during the first 6 years of a mandatory audit within the dutch pancreatic cancer group. Ann Surg 278(2):260–266 35866656 10.1097/SLA.0000000000005628

[21] Wu Y, Peng B, Liu J, Yin X, Tan Z, Liu R et al (2023) Textbook outcome as a composite outcome measure in laparoscopic pancreaticoduodenectomy: a multicenter retrospective cohort study. Int J Surg 109(3):374–382 36912568 10.1097/JS9.0000000000000303 PMC10389643

[22] Canaslan K, Ates Bulut E, Kocyigit SE, Aydin AE, Isik AT (2022) Predictivity of the comorbidity indices for geriatric syndromes. BMC Geriatr 22(1):440 35590276 10.1186/s12877-022-03066-8 PMC9118684

[23] Tan E, Song J, Lam S, D’Souza M, Crawford M, Sandroussi C (2019) Postoperative outcomes in elderly patients undergoing pancreatic resection for pancreatic adenocarcinoma: a systematic review and meta-analysis. Int J Surg 72:59–68 31580919 10.1016/j.ijsu.2019.09.030

[24] Serretta V, Tulone G, Baiamonte D, Muffoletto F, Gesolfo CS (2020) Frailty index vs ASA score: which is better to stratify urologic surgery risk in oncological and non-oncological patients? Eur Urol Open Sci 20:S173

[25] Malleo G, Mazzarella F, Malpaga A, Marchegiani G, Salvia R, Bassi C et al (2013) Diabetes mellitus does not impact on clinically relevant pancreatic fistula after partial pancreatic resection for ductal adenocarcinoma. Surgery 153(5):641–650 23276391 10.1016/j.surg.2012.10.015

[26] Chong E, Ratnayake B, Lee S, French JJ, Wilson C, Roberts KJ et al (2021) Systematic review and meta-analysis of risk factors of postoperative pancreatic fistula after distal pancreatectomy in the era of 2016 International Study Group pancreatic fistula definition. HPB 23(8):1139–1151 33820687 10.1016/j.hpb.2021.02.015

[27] Callery MP, Pratt WB, Kent TS, Chaikof EL, Vollmer CM (2013) A prospectively validated clinical risk score accurately predicts pancreatic fistula after pancreatoduodenectomy. J Am Coll Surg 216(1):1–14 23122535 10.1016/j.jamcollsurg.2012.09.002

[28] Mavroeidis V, Russell T, Clark J, Adebayo D, Bowles M, Briggs C et al (2023) Pancreatoduodenectomy for suspected malignancy: nonmalignant histology confers increased risk of serious morbidity. Ann Royal College Surg England 105(5):446–454 10.1308/rcsann.2022.0055 PMC10149251 35904332

